# A Social Media Content Analysis of Dental Health Information Involving the Use of Miswak (Salvadora persica) Chewing Stick on YouTube™

**DOI:** 10.7759/cureus.64743

**Published:** 2024-07-17

**Authors:** Maram A Alwadi, AlBandary H AlJameel, Falah R Alshammari, Enmanuel A Chavarria, Basil H Aboul-Enein

**Affiliations:** 1 Department of Dental Health, College of Applied Medical Sciences, King Saud University, Riyadh, SAU; 2 Department of Periodontics and Community Dentistry, College of Dentistry, King Saud University, Riyadh, SAU; 3 Department of Dental Public Health and Community Dentistry, College of Dentistry, Hail University, Hail, SAU; 4 Department of Behavioral, Social, and Health Education Sciences, Rollins School of Public Health, Emory University, Atlanta, USA; 5 Faculty of Public Health and Policy, London School of Hygiene & Tropical Medicine, London, GBR

**Keywords:** internet, youtube, dental health, miswak, social media

## Abstract

Background

The widespread availability of Internet access and the rising popularity of social media platforms have facilitated the dissemination of health-related information, including dental health practices. However, assessing the quality and effectiveness of such information remains a challenge, particularly concerning traditional practices such as Miswak (*Salvadora persica*) usage. This study aims to assess the description, use, and effectiveness of the Miswak (*Salvadora persica*) chewing stick posted as video clips on YouTube™ and provide considerations for future interventions.

Methodology

YouTube videos were searched using the terms “Miswak,” “Siwak,” “Salvadora persica,” and “Chewing stick.” Each video’s descriptive features, i.e., title, links, country of origin, upload date, running time, views, comments, likes, and dislikes, were recorded. Content quality was assessed using the DISCERN tool, which rates the reliability, dependability, and trustworthiness of online sources across 16 items. Scores were aggregated for analysis. The statistical analysis examined video features and associations between the speaker, video type, source, and quality, with significance set at a p-value <0.05 using SPSS Statistics Version 20 (IBM Corp., Armonk, NY, USA).

Results

A total of 45 videos were included in the study, with the majority (62%) created by the “other professionals” category. Almost three-quarters (73.3%) of the videos were educational. The quality of the video clips was correlated with the speaker source and category of “other,” revealing that high-quality information was considered such when the source was other than a dentist. Further, we found that a video’s source did not elicit differences in the opinion of the video’s quality.

Conclusions

This social media analysis provides considerations and implications for future research on the potential use of YouTube as a platform for Miswak educational interventions.

## Introduction

According to recent literature, there are more than five billion internet users globally, almost two-thirds (63.1%) of the global population, with 59% using social media [1]. Internet use among Americans is at 93%, with data showing a sharp increase in internet use among people from different age groups, races, genders, and from all income and educational backgrounds [2]. The National Telecommunications and Information Administration in their 2019 Internet Use Survey reported that more households are using the internet for researching health information [3]. Several recent studies indicate that social media may be used as a viable medium to deliver public and dental health-related information to target populations [4-16]. Yet, globally, many face challenges in critically understanding and appraising health information online and on social media; furthermore, formal training or appropriate guidance on the use of online resources for health is needed [17]. This unique skill set is termed eHealth literacy [18]. eHealth literacy encompasses the ability to seek, find, understand, and appraise health information from electronic sources and apply this knowledge to addressing or solving a health problem. Improving eHealth literacy can empower individuals to make informed health decisions and engage effectively with healthcare providers [18].

The internet contains a multitude of video content through different platforms. For example, YouTube™ is a free video-sharing and social media broadcasting platform with millions of users daily [19]. Its extensive reach to communicate health-related messages may be attributed to its accessibility from numerous devices, but its impact on behavior is difficult to assess [13,20,21]. Furthermore, YouTube™ content may be inappropriate for individuals with low levels of eHealth literacy [22]. A recent systematic review cautions that YouTube™ expert reviews of the content should add to the ranking criterion, instead of using the number of views and likes as a basis for quality [23].

Although oral diseases are preventable, they are among the most common untreated non-communicable diseases globally, with the maintenance of oral health and hygiene used as a tool of education by dental care professionals [24]. In Middle Eastern countries, one of the effective natural ways of maintaining oral health is the regular use of Miswak (*Salvadora persica*), which is a chewing stick made out of roots, branches, and stems of the *Salvadora persica* tree [25-31]. Indeed, the World Health Organization (WHO) endorses the use of Miswak to improve oral health [32]. Evidence confirms the beneficial effects of using Miswak as it has antifungal, anticariogenic, antiplaque, and antioxidant effects [25,33].

Almas and Al-Lafi suggest that to achieve the desired cleaning effect of Miswak, the tip intended for brushing should be dipped in water for two to five minutes to soften it slightly before use [34]. The tip should then be chewed until hairy-like structures form, which will then serve as a toothbrush. Furthermore, it is recommended to bite Miswak equally along all the tooth surfaces before using it across tooth and gum areas. Cleaning the contact surface of teeth in this way can be beneficial as it helps remove plaque and food particles from between the teeth, reducing the risk of cavities and gum disease. For the best results, it is wise to ensure the areas used for Miswak are always uncontaminated for the Miswak tip to produce an adequate amount of benzyl isothiocyanate every time it is utilized in the mouth. The usage of the same endpiece multiple times gradually reduces the amount of benzyl isothiocyanate emitted [35]. Niazi et al. (2016) recommend using Miswak regularly to help reduce plaque accumulation and increase dental hygiene [36]. Utilizing Miswak is a cost-effective and culturally adopted dental health behavior practiced in many countries throughout Asia, Africa, South America, and the Middle East [32,33,37]. More recently, the use of Miswak has spread globally [33].

As such, due to its expansive reach, there is high potential for the dissemination of Miswak educational videos that leverage YouTube™ to provide credible instructional information on the use of Miswak and other oral hygiene measures. As mentioned above, Miswak is an excellent choice as an alternative oral hygiene instrument [36]. However, there is a need to master how to handle and use it properly to achieve the best results effectively.

Therefore, the purpose of this study is to assess the description, use, and quality of Miswak (*Salvadora persica*) chewing sticks posted as video clips on YouTube™ and provide considerations for future interventions. This article builds on prior research examining the potential of YouTube™ as a resource for health promotion and explores an existing gap in the literature on the description, use, and effectiveness of information related to Miswak.

## Materials and methods

For the scientific premise, the methodology reported in other recently published social media content analyses on YouTube™ [20,38-40] was followed in this study. Due to its observational nature and use of publicly accessible data, this study was exempt from ethical approval. To ensure the anonymity of the data, YouTube™ links were intentionally omitted from the study.

YouTube™ video search procedures

A YouTube™ video search was conducted in August 2023, using the search terms “Miswak,” “Siwak,” “Salvadora persica,” and “Chewing stick.” Previous studies indicate that 95% of users who search on YouTube™ are more likely to watch videos from the first 60 results of their search terms [41-47]. To ensure comprehensive coverage, the first 100 videos for each search term were viewed and evaluated. The YouTube™ settings were adjusted to rank videos from the highest to the least viewed, and the top 100 rated videos were selected from the search results. Inclusion criteria were applied to determine which videos would be included in the analysis.

Video selection and exclusion criteria

**Figure 1:**
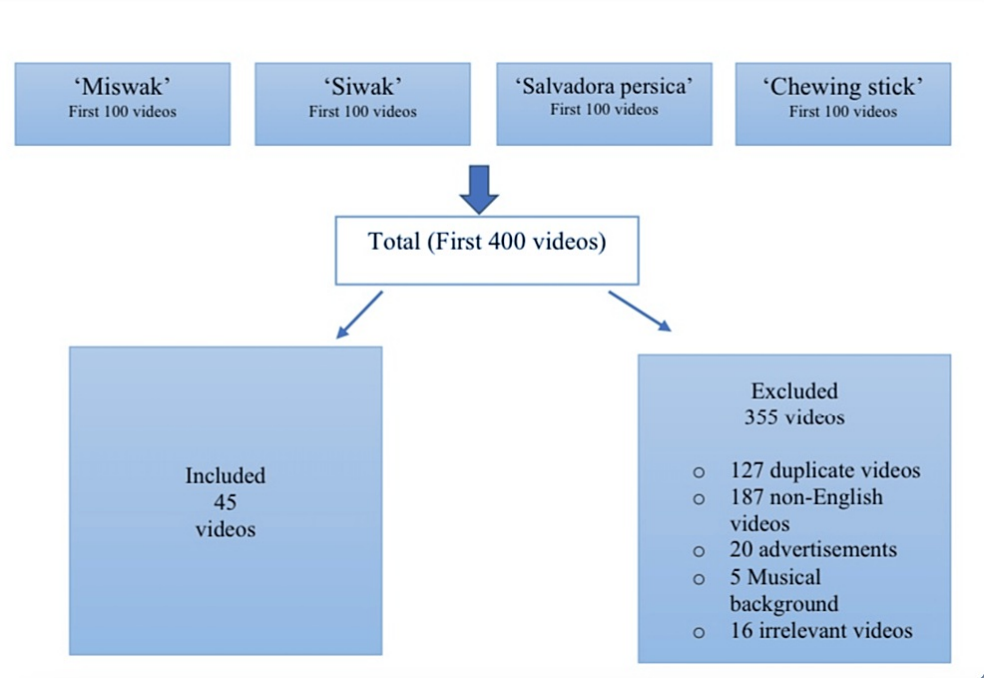
Detailed study workflow.

During the video selection process, various exclusion criteria were applied. Videos that were not in English and/or Arabic, deemed irrelevant, lacking sound or headings, duplicates, cartoon-related, with a musical background, commercials, lacking information, or longer than 15 minutes were excluded. Videos longer than 15 minutes were excluded based on insights from recent literature on YouTube-related health information-seeking studies, which indicate that viewers’ attention tends to peak at around 15 minutes and diminish thereafter [48]. This criterion was applied to ensure that selected videos align closely with typical viewer engagement patterns on the platform. The methodology closely followed the approach outlined previously [48], and the screening process of the videos was guided by the stages outlined in Figure 1.

Video sources and evaluation

The selected videos were evaluated by two dental professionals (MW, AJ) independently and in a blinded manner, ensuring an unbiased assessment. They reviewed the videos and collected data accordingly. Descriptive characteristics of each video, including title, links, country of origin, upload date, running time, number of views, comments, likes, and dislikes, were recorded during analysis.

Evaluators (MW, AJ) categorized the video’s speakers into the following six groups: general dentists, dental specialists, dental hygienists, researchers, influencers/actors, not available (N/A), and others. The video type was categorized into two categories (educational and experiential). The video source was reported according to the type of source (for-profit organization, non-profit organization, and individual user) (Table 1). Any disagreements or discrepancies were resolved through discussion, and if not resolved, by a third member of the research team (BA). Videos were further categorized by dental professionals in terms of accuracy and reliability using DISCERN (Appendices) which was created for use in healthcare by both health professionals and the general public to evaluate the quality and reliability of health information on the internet [49].

**Table 1:** Details of all videos about dental health information involving the use of Miswak (Salvadora persica) chewing sticks on YouTube included in the study. NA = not available

YouTube #	Country of origin	Upload date	Running time	Views	Comments	Likes	Dislikes	Speaker	Video type	Video source
1	India	07/08/2016	02:52 minutes	74,998	49	1.1K	0	NA	Educational	Individual user
2	South America	02/08/2021	00:50 minutes	1,183	Turned off	0	0	Other, e-commerce website	Educational	Profit organization
3	Washington	23/01/2016	08:06 minutes	142,079	113	1.5K	0	Other, holistic health and healing expert	Educational	Profit organization
4	India	09/06/2018	02:48 minutes	25,625	25	275	0	Influencer	Educational	Individual user
5	United Arab Emirates	04/04/2022	02:50 minutes	396	3	21	0	Dentist	Educational	Non-profit organization
6	United States	22/02/2019	06:48 minutes	11,628	147	659	0	Influencer	Experience	Individual user
7	Pakistan	03/10/2020	03:51 minutes	21,581	57	384	0	Researcher	Educational	Individual user
8	United States	10/09/2015	02:09 minutes	118,622	254	1.6K	0	Other, e-commerce website	Educational	Profit organization
9	United States	15/07/2015	02:15 minutes	18,716	0	0	0	Other, e-commerce website	Experience	Profit organization
10	United States	21/04/2019	02:49 minutes	419,645	225	11K	0	Other, e-commerce website	Educational	Profit organization
11	United States	10/08/2015	11:32 minutes	16,497	114	72	0	Other, Emily Wolff	Experience	Individual user
12	United States	17/07/2015	05:11 minutes	117,535	139	1K	0	Influencer	Experience	Individual user
13	United States	05/01/2021	01:00 minutes	27,183	85	1K	0	Other, TADÁS WELLNESS	Educational	Individual user
14	California	29/03/2019	01:53 minutes	80	1	1	0	Influencer	Experience	Individual user
15	Pakistan	18/09/2021	03:14 minutes	106	5	5	0	NA	Educational	Individual user
16	United States	08/04/2016	09:04 minutes	1,327	1	10	0	Other, seller	Experience	Individual user
17	India	03/04/2018	02:04 minutes	74	0	0	0	NA	Educational	Non-profit organization
18	NA	06/05/2018	03:44 minutes	31	0	2	0	Other, Margaret Kaliczynski	Experience	Individual user
19	United Kingdom	29/04/2021	02:28 minutes	74	1	8	0	Other, dietitian and a personal trainer	Educational	Individual user
20	Saudi Arabia	14/09/2022	01:20 minutes	57	0	3	0	Periodontist	Educational	Individual user
21	Egypt	18/07/2019	00:31 minutes	89,941	67	1.7K	0	Other, a religious supervisor on a TV channel	Educational	Profit- organization
22	NA	05/06/2022	00:40 minutes	113	0	4	0	NA	Educational	Profit organization
23	United States	03/09/2020	03:29 minutes	152	0	8	0	Influencer	Experience	Individual user
24	United Kingdom	17/06/2021	01:20 minutes	442	0	12	0	Influencer	Educational	Individual user
25	Pakistan	30/05/2018	01:08 minutes	22,264	0	448	0	Influencer	Educational	Profit-organization
26	India	13/02/2022	06:53 minutes	456	0	9	0	Influencer	Educational	Individual user
27	Pakistan	18/04/2021	05:13 minutes	202	0	26	0	Other, Usk Fam	Educational	Individual user
28	Pakistan	22/11/2021	1:00 minutes	9,067	13	474	0	Others, ArabicMclovin	Educational	Individual user
29	United Kingdom	05/05/2015	6.37 minutes	11,575	15	82	0	Others, Natural Spa Supplies Ltd.	Experience	For-profit organization
30	NA	12/08/2021	6.09 minutes	271	1	5	0	Others, harrymetsally2	Experience	Individual user
31	India	30/04/2020	9.49 minutes	816	11	36	0	Others, Feel Good Living	Educational	Individual user
32	United Kingdom	21/12/2018	8.08 minutes	5,485	34	78	0	Others, Natural Spa Supplies Ltd.	Educational	For-profit organization
33	United States	03/07/2020	4.04 minutes	7,439	65	323	0	Others, Angelic Africa	Educational	Individual user
34	United Kingdom	15/09/2008	4.29 minutes	3,335	0	12	0	Others, Natural Museum	Experience	Natural Museum
35	Chicago	19/09/2021	4.51 minutes	3+ million	19,626	48,2000	0	Dental student	Educational	Individual user
36	Canada	05/08/2020	14.24 minutes	31,773	149	539	0	Others, Brian Eng	Educational	Individual user
37	United States	05/01/2020	4.32 minutes	45,537	110	671	0	Dentist	Educational	Individual user
38	NA	27/09/2021	1:00 minutes	287	0	5	0	Others, Political Tech	Educational	Individual user
39	Australia	10/08/2022	1:00 minutes	177	0	1	0	Others, ThatRandomo	Educational	Individual user
40	United States	23/08/ 2018	14.23 minutes	273	2	12	0	Others, StrawBoys	Educational	Individual user
41	United States	27/05/2017	6.16 minutes	77	0	4	0	Others, CommonWealth Herbs	Educational	CommonWealth Centre, for-profit organization
42	United States	12/08/2016	7.06 minutes	141	0	3	0	Others, Natural Needs Product	Educational	Natural Needs Products, for-profit organization
43	Canada	07/09/2022	1.00 minutes	14	0	0	0	Others, Witness TV	NA	Individual user
44	Kuwait	07/11/2019	7.28 minutes	1,587	0	12	0	Others, Arabic Lessons with Hazem	Educational	Individual user
45	United States	03/09/2020	1.27 minutes	17	0	1	0	Others, Wiki4All	Educational	Not for profit

DISCERN tool

DISCERN is a standardized questionnaire with 16 items on a Likert-type rating scale ranging from 1 to 5, with 1 being a definite no and 5 representing a definite yes. Any ranking in the middle (e.g., 2, 3, or 4) indicates that some of the elements requested by the question are present to some level. These questions are divided into three categories. Section 1 (questions 1 to 8) evaluates a website’s reliability, trustworthiness, and dependability; section 2 (questions 9 to 15) concentrates on the quality of the information; and section 3 (question 16) assesses the overall quality rating on a continuous rating scale for the online media, with a rating of 1-2 considered a low-quality video with “serious shortcomings,” a score of 3 indicating moderate quality video with “some limitations,” and a score of 4-5 indicating a high quality or a “useful source” video. The rating given for question 16 is separate from the ratings given for the first 15 questions.

The total scores for the 16 questions were calculated. The DISCERN tool contains three predetermined cut-off points that define the video quality level, with a maximum score of 80 and a minimum score of 16. Low quality ranges from 16 to 37.6, medium ranges from 37.7 to 58.9, and high ranges from 59 to 80. The reliability of the videos was determined by taking the average of the first eight questions from (1 to 8), and from 9 to 15 questions were averaged to evaluate the quality of the information.

Each video was independently evaluated by three specialists in dentistry and dental health information related to Miswak (*Salvadora persica*) (blind for review). Specialists first familiarized themselves with the DISCERN tool using sample videos to ensure evaluation consistency. The average of their assessments was then calculated and documented for each video. Any discrepancies were resolved through discussion or with input from a fourth team member to uphold a rigorous and unbiased assessment process. The DISCERN tool used for evaluation is available in the Appendices.

Statistical analysis

The statistical analysis of the data was performed using the SPSS Statistics Version 20 (IBM Corp., Armonk, NY, USA). The features of videos (number of views, duration in minutes, number of comments, number of likes, and number of dislikes) were represented by mean ± SD as well as by minimum and maximum counts. The normal distribution of the data was evaluated using the Kolmogorov-Smirnov test. Further, chi-square tests were used to examine the associations between the speaker, message type, source of information, and video quality using the DISCERN tool because the data were categorical. The correlation between variables of quality assessments (performed with the DISCERN tool) and the characteristics of the videos was tested using Spearman’s rho because data were not normally distributed. Results were considered significant at a p-value <0.05.

## Results

As shown in Table 1, a total of 45 videos were selected based on the inclusion and exclusion criteria and evaluated accordingly. Data regarding videos’ country of origin, upload date, running time, number of views, comments, likes and dislikes, type of speaker, video type, and video source are presented in detail in Table 1.

The total number of views per video had a large variability, ranging from 14 to 3,000,000 views (Table 2). The shortest video was approximately 31 seconds, while the longest one was just above 14 minutes (Table 2). Regarding the speaker type, the “Other professionals” category represented 62.20% (n = 28 out of 45) of the speakers in the videos, followed by influencers at 17.80% (n = 8 out of 45). In terms of categorization of video type, the majority (73.30%, n = 33 out of 45) were educational, and individual users made up the highest percentage in the video source category (64.40%, n = 29 out of 45) (Table 3).

**Table 2:** Characteristics of videos about dental health information involving the use of Miswak (Salvadora persica) chewing sticks on YouTube included in the study (N = 45). Data are displayed as mean, standard deviation (SD), and minimum and maximum values.

Variables	Mean	SD	Minimum	Maximum
Number of views	69,388.63	446,876.45	14	3,000,000
Videos length in minutes	261.60	216.76	0.31	14.24
Number of comments	473.60	2,920.64	0	19,626
Number of likes	11,224.56	71,793.31	0	482,000

**Table 3:** Speakers, video types, and video sources about dental health information involving the use of Miswak (Salvadora persica) chewing sticks on YouTube (N = 45).

Speaker		N	%
	Dental student	1	2.20
	Dentist	2	4.40
	Influencer	8	17.80
	NA	4	8.90
	Other	28	62.20
	Periodontist	1	2.20
	Researcher	1	2.20
	Total	45	100.00
Video type		N	%
	Educational	33	73.30
	Experience	11	24.40
	NA	1	2.20
	Total	45	100.00
Video source		N	%
	Individual user	29	64.40
	Natural museum	1	2.20
	Non-profit organization	3	6.70
	Profit organization	12	26.70
	Total	45	100.00

Table 4 shows the quality assessments of included videos using the DISCERN tool. The overall DISCERN scores ranged from 16 to 60.8, with the highest reliability score being 4.25 and the lowest 1.25. The quality of information scores varied from 1.0 to 3.6, and the overall quality ratings ranged from 1 to 3.8. The total DISCERN scores categorized the videos into the following three quality levels: low quality (16-37.6), medium quality (37.7-58.9), and high quality (59-80). Most videos fell into the low (23 videos) and medium (15 videos) quality categories, with only a few (3 videos) achieving high-quality ratings. This assessment underscores the variability in the quality and reliability of health-related content available online.

**Table 4:** Quality assessments of the included videos utilizing the DISCERN tool. *: DISCERN cutoff: low quality ranges from 16 to 37.6; medium quality ranges from 37.7 to 58.9; high quality ranges from 59 to 80.

YouTube #	DISCERN Q1	Q2	Q3	Q4	Q5	Q6	Q7	Q8	Average (Q1 to 8): reliability	Q9	Q10	Q11	Q12	Q13	Q14	Q15	Average (Q9 to 15): quality of the Info.	Q16 overall quality	Total score *
1	5	5	4	5	5	3	1	1	3.7	4	4	1	1	4	1	1	2.3	3	48
2	5	5	5	1	1	2	1	1	2.6	5	5	1	1	3	1	1	2.4	2.5	40.5
3	5	5	2	1	1	2	2	1	2.4	3	2	1	1	1	2	1	1.6	2	32
4	5	5	5	1	1	3	1	2	2.9	5	5	3	3	4	3	1	3.4	3.15	50.15
5	5	5	4	1	1	4	1	1	2.8	5	3	2	1	2	2	1	2.3	2.6	40.6
6	5	5	5	5	3	3	5	3	4.25	5	4	2	2	2	5	2	3.1	3.7	59.7
7	5	5	5	5	5	3	4	2	4.25	5	5	1	3	3	5	1	3.3	3.8	60.8
8	5	5	5	5	5	3	3	1	4	5	5	1	3	3	3	1	3	3.5	56.5
9	5	5	1	1	1	1	1	1	2	5	5	3	3	3	3	1	3.3	2.65	41.65
10	5	5	1	1	1	1	1	1	2	5	1	1	1	1	2	1	1.7	1.9	29.9
11	3	3	1	1	1	1	1	1	1.5	3	2	1	1	2	1	1	1.6	1.6	24.6
12	5	5	3	1	1	3	1	1	2.5	5	4	1	1	3	3	1	2.6	2.6	40.6
13	5	5	5	5	5	1	1	1	3.5	3	5	1	1	2	2	1	2.1	2.8	45.8
14	5	3	1	1	1	3	1	1	2	3	3	1	1	2	1	1	1.7	1.85	29.85
15	5	5	3	2	1	3	5	1	3.12	3	5	1	2	3	3	1	2.6	2.9	45.9
16	5	5	1	1	1	1	1	1	2	3	3	1	2	3	1	2	2.1	2.05	33.05
17	5	5	3	1	1	3	1	1	2.5	3	5	1	1	3	2	1	2.3	2.4	38.4
18	4	3	1	1	1	1	1	1	1.6	2	3	1	1	2	2	1	1.7	1.7	26.7
19	5	5	5	4	4	5	3	1	3.6	4	5	1	3	4	5	3	3.6	3.6	60.6
20	5	5	3	1	1	5	1	2	2.9	5	5	5	3	3	2	1	3.4	3.15	50.15
21	5	5	1	1	1	2	1	1	2.1	2	2	1	1	2	1	1	1.4	1.8	28.8
22	3	3	3	3	3	3	1	1	2.5	3	5	1	1	2	2	1	2.1	2.3	37.3
23	5	5	1	1	1	1	1	1	2	2	3	1	2	2	1	1	1.7	1.9	29.9
24	5	5	5	4	3	5	3	5	3.75	3	4	1	2	3	1	1	2.1	2.3	52.3
25	5	5	1	1	1	2	1	1	2.1	3	5	3	2	4	1	1	2.7	2.4	38.4
26	5	5	3	1	1	3	1	3	2.75	5	5	3	3	5	2	1	3.4	3.1	49.1
27	5	5	4	1	1	3	1	1	2.6	3	5	1	3	3	1	1	2.4	2.5	40.5
28	4	4	5	3	1	3	1	1	2.75	4	4	1	1	1	1	3	2.14	2.5	39.5
29	3	3	1	1	1	3	1	1	1.75	1	1	1	1	1	1	1	1	1.5	22.5
30	3	3	3	1	1	1	1	1	1.75	2	3	1	1	1	3	1	1.7	1.7	27.7
31	5	5	3	1	1	2	1	1	2.38	3	2	2	2	1	4	1	2.14	2.3	36.3
32	5	4	4	5	5	4	2	2	3.87	3	3	1	1	3	5	3	2.71	3.29	53.29
33	3	3	4	1	1	1	1	1	1.86	2	1	1	1	1	3	1	1.43	1.65	26.65
34	2	2	2	1	1	1	1	1	1.36	1	1	1	1	1	1	1	1	1.18	19.18
35	5	5	5	1	1	4	1	1	2.86	3	1	1	1	1	1	3	1.57	2.3	36.3
36	3	3	5	2	1	3	1	1	2.38	2	2	1	1	1	4	1	1.71	2	33
37	5	5	5	4	1	4	2	2	3.5	3	4	2	2	3	5	3	3.14	3.3	53.3
38	2	2	3	1	1	2	1	1	1.63	1	1	1	1	1	1	1	1	1.3	21.3
39	1	1	3	1	1	1	1	1	1.25	1	1	1	1	1	1	1	1	1.1	18.1
40	4	4	4	4	3	3	1	1	3	3	3	1	1	1	3	2	2	2.5	40.5
41	3	3	3	1	1	3	1	1	2	2	2	1	1	2	2	1	1.56	1.8	28.8
42	5	5	5	5	1	4	3	2	3.75	2	3	1	1	2	2	3	2	2.9	46.9
43	1	1	1	1	1	1	1	1	1	1	1	1	1	1	1	1	1	1	16
44	4	4	4	3	1	3	1	1	2.63	3	3	1	1	1	3	1	1.86	2.2	36.2
45	5	5	5	5	4	4	4	1	4.13	3	3	1	3	2	2	1	2.14	3.1	51.1

Regarding the quality assessment of videos relevant to the speaker (Table 5), the videos from the “Other professionals” category had the largest percentage at the “low total score” (37.8%, n = 17 out of 45 videos). A significant association (p = 0.021) was found for assessed video quality between the total score subcategories. A significant association (p = 0.023) was also found among subcategories of “Quality of the Info.” No significant associations were found among subcategories of reliability and overall quality (p = 0.477 and 0.112, respectively).

**Table 5:** Quality assessment of videos utilizing the DISCERN tool in correlation to the speaker. *: NA = not available; **: level of significance = p-value <0.05. Statistical tests used: chi-square tests (X²) for the association between video quality assessments and speaker types.

	Total score			Reliability			Quality of the information			Overall quality		
	Low N %	Medium N %	High N %	Low N %	Medium N %	High N %	Low N %	Medium N %	High N %	Low N %	Medium N %	High N %
Dental student	1	0	0	0	1	0	1	0	0	0	1	0
	2.2%	0.0%	0.0%	0.0%	2.2%	0.0%	2.2%	0.0%	0.0%	0.0%	2.2%	0.0%
Dentist	0	2	0	0	1	1	0	1	1	0	1	1
	0.0%	4.4%	0.0%	0.0%	2.2%	2.2%	0.0%	2.2%	2.2%	0.0%	2.2%	2.2%
Influencer	2	5	1	0	6	2	2	3	3	2	3	3
	4.4%	11.1%	2.2%	0.0%	13.3%	4.4%	4.4%	6.7%	6.7%	4.4%	6.7%	6.7%
Other	17	10	1	9	12	7	15	11	2	13	11	4
	37.8%	22.2%	2.2%	20.0%	26.7%	15.6%	33.3%	24.4%	4.4%	28.9%	24.4%	8.9%
Periodontist	0	1	0	0	1	0	0	0	1	0	0	1
	0.0%	2.2%	0.0%	0.0%	2.2%	0.0%	0.0%	0.0%	2.2%	0.0%	0.0%	2.2%
Researcher	0	0	1	0	0	1	0	0	1	0	0	1
	0.0%	0.0%	2.2%	0.0%	0.0%	2.2%	0.0%	0.0%	2.2%	0.0%	0.0%	2.2%
NA *	1	3	0	0	2	2	0	4	0	0	4	0
	2.2%	6.7%	0.0%	0.0%	4.4%	4.4%	0.0%	8.9%	0.0%	0.0%	8.9%	0.0%
P-value **	0.021			0.477			0.023			0.112		

Regarding assessed video quality relevant to the video type (Table 6), the educational category had a greater share of the tally fall in the “medium total score” (42.2% of the videos). A significant association (p = 0.019) was found for the reliability of assessed videos with the educational video type with the highest percentage of total scores medium quality (40%) and high quality (26.7%). No significant associations were found within the subcategories “Quality of the Info” or “Overall quality.”

**Table 6:** Quality assessments of videos utilizing the DISCERN tool in correlation to the video type. *: NA = not available; **: level of significance = p-value <0.05.

Video type	Total score			Reliability			Quality of the information			Overall quality		
	Low N %	Medium N %	High N %	Low N %	Medium N %	High N %	Low N %	Medium N %	High N %	Low N %	Medium N %	High N %
Educational	12	19	2	3	18	12	10	17	6	7	17	9
	26.7%	42.2%	4.4%	6.7%	40.0%	26.7%	22.2%	37.8%	13.3%	15.6%	37.8%	20.0%
Experience	8	2	1	5	5	1	7	2	2	7	3	1
	17.8%	4.4%	2.2%	11.1%	11.1%	2.2%	15.6%	4.4%	4.4%	15.6%	6.7%	2.2%
NA *	1	0	0	1	0	0	1	0	0	1	0	0
	2.2%	0.0%	0.0%	2.2%	0.0%	0.0%	2.2%	0.0%	0.0%	2.2%	0.0%	0.0%
P-value **	0.174			0.019			0.199			0.066		

Regarding assessed video quality relevant to the video source (Table 7), “individual user” had its greatest share of scores falling within “low quality” in the total score tally. No significant associations were found among any video source subcategories compared in our analyses.

**Table 7:** Quality assessments of videos utilizing the DISCERN tool in correlation to the video source. *: Level of significance = p-value <0.05.

Video source	Total score			Reliability			Quality of the information			Overall quality		
	Low N %	Medium N %	High N %	Low N %	Medium N %	High N %	Low N %	Medium N %	High N %	Low N %	Medium N %	High N %
Individual user	14	12	3	7	13	9	12	10	7	9	13	7
	31.1%	26.7%	6.7%	15.6%	28.9%	20.0%	26.7%	22.2%	15.6%	20.0%	28.9%	15.6%
Natural museum	1	0	0	1	0	0	1	0	0	1	0	0
	2.2%	0.0%	0.0%	2.2%	0.0%	0.0%	2.2%	0.0%	0.0%	2.2%	0.0%	0.0%
Non-profit organization	0	3	0	0	2	1	0	3	0	0	2	1
	0.0%	6.7%	0.0%	0.0%	4.4%	2.2%	0.0%	6.7%	0.0%	0.0%	4.4%	2.2%
Profit organization	6	6	0	1	8	3	5	6	1	5	5	2
	13.3%	13.3%	0.0%	2.2%	17.8%	6.7%	11.1%	13.3%	2.2%	11.1%	11.1%	4.4%
P-value *	0.400			0.347			0.279			0.672		

Preliminary correlations between video characteristics and assessed video quality are summarized in Table 8. Significant correlations were found between views and length in minutes (significant), number of likes (significant), and total score (negative [significant]). Significant correlations were also found between length in minutes and the comments category. Other significant correlations are shown in Table 8.

**Table 8:** Correlation (Spearman’s rho) between quality assessment variables (utilizing the DISCERN tool) and video characteristics. **: Correlation is significant at the 0.01 level (two-tailed); *: Correlation is significant at the 0.05 level (two-tailed).

		Views	Length in minutes	Comments	Likes	Dislikes	Overall quality	Reliability	Information quality	Total score
Spearman’s rho	Views	1.000	0.394^**^	0.208	0.341^*^	.	-0.145	-0.132	-0.280	-0.352^*^
	Length in minutes	-	1.000	0.321^*^	0.267	.	-0.044	0.071	-0.058	-0.119
	Comments	-	-	1.000	0.833^**^	.	0.036	0.233	-0.021	0.038
	Likes	-	-	-	1.000	.	0.076	0.238	-0.011	0.059
	Dislikes	-	-	-	-	.	.	.	.	.
	Overall quality	-	-	-	-	.	1.000	0.675^**^	0.875^**^	0.800^**^
	Reliability	-	-	-	-	-	-	1.000	0.637^**^	0.724^**^
	Information quality	-	-	-	-	-	-	-	1.000	0.870^**^
	Total score	-	-	-	-	-	-	-	-	1.000

## Discussion

To our knowledge, this is the first study to assess video clips on YouTube™ on their description, use, and effectiveness of Miswak (*Salvadora persica*) chewing sticks within the published literature. The current assessment of YouTube™ video clips relevant to Miswak builds on prior research examining the potential of YouTube™ as a resource for dental health promotion and explores an existing gap in the dental research literature. Previous studies examining dental and oral health education through social media suggest that YouTube™ and other social media websites offer interactive educational possibilities for dentistry and dental public health; however, dental health professionals should play a more proactive role in educative information given on social media, particularly YouTube™, and become more involved with monitoring the content disseminated through these kinds of platforms [9,16,50,51]. The ease and popularity of accessing YouTube™ not only through desktops but also on mobile devices, smartphones, and tablets make it a wide-reaching tool in supplementing dental and oral health education worldwide. Consideration should be given to the quality of the videos themselves given that they may influence whether a speciﬁc educational goal is met or not.

Our concise presentation of descriptive statistics on YouTube™ videos relevant to Miswak assists future interventionists in dental care with understanding a broad scope of characteristics, including speaker sources, video types, and video sources.

The quality of the video information was correlated with the speaker source “Other” (p = 0.023). This finding is interesting in that we would expect a speaker source such as “Dentist” to be considered high quality and would make for a greater percentage of appearance in videos. Yet, our assessment found the category “Other” to make a greater number of appearances in the selected video clips. These findings agree with that of a previous study where patients mostly made appearances in videos describing orthognathic surgery and not orthodontics or surgeons as expected [52].

The reliability of videos was significantly correlated with the clips categorized as “educational” (p = 0.019). Greater tallies for educational videos were in the “medium” and “high” categories. This finding is of interest to further investigate as it is an expected finding but yet not previously analyzed in prior similar dental and YouTube™ examinations [39,50,53].

One of the major findings in our analysis is the suggestion that a video’s source does not muster differences in opinion of the video’s quality. Research by Uzel et al. (2023) investigating YouTube™ as an information source in pediatric dentistry similarly concluded that a video’s source showed no statistical correlation with other variables related to video quality [54]. Indeed, this warrants further research to shed light on the consistency of this finding.

Finally, in investigating possible correlations between DISCERN variables (i.e., overall quality, reliability, information quality, total score) and those variables on video characteristics (i.e., views, length in minutes, comments, likes, dislikes) of interest is an inverse correlation between views and all variables of DISCERN. Although only total score and views were statistically significant, this analysis warrants a second look in future efforts. A previous study by Şen et al. (2022) reported that these two sets of variables might not always result in expected outcomes [40]. Typically, one might assume that greater views might be correlated with greater perceived video quality; yet, the current work and prior efforts in the literature convey a need for further exploration of these variables.

Implications of our findings for future work suggest that the development of videos on Miswak would benefit from being more educational and not communicated as experiential narratives. Thus, professionals such as health educators might be best positioned to develop educational content that will resonate with audiences. Further, a thorough understanding of YouTube’s potential for dissemination of Miswak dental health education can significantly bolster public health efforts aimed at improving overall oral health. Moreover, awareness of Miswak may also influence future research on potential positive effects related to cardiac health and other illnesses linked to oral health. Exploring the potential of other social media outlets, such as TikTok, and assessing oral health-related educational videos posted on them is also suggested in future research.

While this study achieved its research objectives by providing a comprehensive analysis of YouTube™ videos related to Miswak (*Salvadora persica*) chewing sticks, several limitations should be acknowledged. First, the restriction to videos in English and Arabic may have excluded relevant content in other languages, potentially limiting the generalizability of the findings. Second, the reliance on publicly accessible data from YouTube™ means that videos set to private or with restricted access were not captured, which might have led to the omission of pertinent information. Additionally, despite the rigorous evaluation process involving two independent dental professionals and the use of the DISCERN tool, some degree of subjectivity in the assessment of videos may still be present. The study also focused solely on YouTube™, excluding other social media platforms that might offer valuable insights. Despite these limitations, the study offers important insights into the potential of YouTube™ as a resource for dental health promotion and highlights areas for future research and intervention.

## Conclusions

The study findings suggest that YouTube™ can be an effective platform for disseminating educational content about Miswak, regardless of the video’s origin. The study highlights the need for dental professionals to engage more actively in creating and curating content on social media platforms to enhance the quality of dental health information and oral health education available to the public. Future research should explore the broader implications of these findings and examine the potential of other social media platforms such as X (formerly known as Twitter), Instagram, and Facebook for similar educational interventions in oral health.

## Appendices

**Table 9:** DISCERN tool variables. From http://www.discern.org.uk/discern_instrument.php. Notes: *: Section 1 (questions 1 to 8) evaluates a website’s reliability, trustworthiness, and dependability. **: Section 2 (questions 9 to 15) concentrates on the quality of the information. ***: Section 3 (question 16) assesses the overall quality rating on a continuous rating scale for the online media.

Question number	What is investigated?
1*	Are the aims clear?
2	Does it achieve its aims?
3	Is it relevant?
4	Is it clear what sources of information were used to compile the publication (other than the author or producer)?
5	Is it clear when the information used or reported in the publication was produced?
6	Is it balanced and unbiased?
7	Does it provide details of additional sources of support and information?
8	Does it refer to areas of uncertainty?
9**	Does it describe how each treatment works?
10	Does it describe the benefits of each treatment?
11	Does it describe the risks of each treatment?
12	Does it describe what would happen if no treatment is used?
13	Does it describe how the treatment choices affect the overall quality of life?
14	Is it clear that there may be more than one possible treatment choice?
15	Does it provide support for shared decision-making?
16***	Based on the answers to all of the above questions, rate the overall quality of the publication as a source of information about treatment choices
